# Effects of School-Based Preventive Measures on COVID-19 Incidence, Hong Kong, 2022

**DOI:** 10.3201/eid2909.221897

**Published:** 2023-09

**Authors:** Tim K. Tsang, Xiaotong Huang, Min Whui Fong, Can Wang, Eric H.Y. Lau, Peng Wu, Benjamin J. Cowling

**Affiliations:** The University of Hong Kong, Hong Kong, China (T.K. Tsang, X. Huang, M.W. Fong, C. Wang, E.H.Y. Lau, P. Wu, B.J. Cowling);; Laboratory of Data Discovery for Health Limited, Hong Kong Science and Technology Park, Hong Kong (T.K. Tsang, E.H.Y. Lau, P. Wu, B.J. Cowling)

**Keywords:** COVID-19, respiratory infections, severe acute respiratory syndrome coronavirus 2, SARS-CoV-2, SARS, coronavirus disease, zoonoses, viruses, coronavirus, school closures, public health, Hong Kong

## Abstract

We show that school closures reduced COVID-19 incidence rates in children by 31%–46% in Hong Kong in 2022. After school reopening accompanied by mask mandates, daily rapid testing, and vaccination requirements, school-reported cases correlated with community incidence rates. Safe school reopening is possible when appropriate preventive measures are used.

After 2 years of minimal incidence of SARS-CoV-2 infections in Hong Kong, the Omicron BA.2 variant began to spread in January 2022. The resulting 5th COVID-19 wave in Hong Kong’s population of 7.3 million persons resulted in >1 million cases and >9,000 deaths during February–April 2022, despite high overall vaccine coverage (*1*). After a low point of <200 cases/day in mid-May, the number of cases resurged, resulting in a 6th wave beginning in June 2022.

Schools in Hong Kong were intermittently closed throughout the 5th wave, and online learning began in February 2022. The summer holiday (conventionally 6 weeks during mid-July–August) was rescheduled to March and April, with a shorter 2-week summer holiday at the end of August. Schools resumed in-person learning in May 2022, and a range of public health and social measures were imposed to reduce COVID-19 transmission risk among staff and students (Table 1; Appendix Tables 1, 2), including mask wearing, requiring negative results of daily self-administered rapid antigen tests (RAT) (Appendix Table 3) for staff and students before entering school, reducing class sizes and lesson durations, and fulfilling certain vaccination requirements.

**Table 1:** Summary of territorywide preventive measures implemented during the 5th and 6th waves of the COVID-19 outbreak in Hong Kong evaluated in study of effects of school-based preventive measures on COVID-19 incidence, 2022

Preventive measures	Focus	Period
Masks		
A person must wear a mask at all times when entering or attending school.	School staff, students	2020 Jan 23–2023 Feb 28
School closure		
Suspend face-to-face classes and on-campus activities	Kindergarten and primary school students	2022 Jan 14–2022 Apr 18
	Students in secondary schools	2022 Jan 24–2022 Apr 28
Allow some mask-wearing activities on a half-day basis	Kindergarten, primary school, and secondary school students	2022 May 19–2022 Oct 31
Resume half-day nonacademic extracurricular activities for those who received 2 vaccine doses >14 d apart	Students in primary schools	2022 Oct 25–2023 Feb 14
Resume half-day nonacademic extracurricular activities for those who received 3 vaccine doses >14 d apart	Students in secondary schools	2022 Oct 1–2023 Jan 31
Resume whole-day face-to-face classes if >90% of vaccination- eligible students (entire school or at individual class level) received >2 vaccine doses >14 d apart	Students in secondary schools	2022 Nov 1–2023 Jan 31
Resume whole-day face-to-face classes if >70% of vaccination- eligible students (entire school or at individual class level) received >2 vaccine doses >14 d apart	Students in primary schools	2022 Dec 1–2023 Feb 14
Resume whole-day face-to-face classes	Students in secondary schools	Beginning 2023 Feb 1
Resume whole-day face-to-face classes	Students in primary schools	Beginning 2023 Feb 15
COVID-19 tests		
Daily rapid antigen test result is required before returning to school for work or lessons	School staff and students	2022 Apr 19–2023 Mar 15
Vaccine pass		
A valid vaccine pass is required for school entry	School staff, students12–17 years of age	2022 Feb 23–2022 Dec 29
Vaccination		
>1 dose	Students 5–11 years of age	2022 Sep 30–2022 Nov 29
	School staff, students 12–17 years of age	2022 Feb 24–2022 Jun 29
>2 doses	Students 5–11 years of age	2022 Nov 30–2023 Feb 15
	School staff, students 12–17 years of age	2022 Jun 30–2022 Nov 29
>3 doses	School staff, students 12–17 years of age	2022 Nov 30–2023 Feb 1

School closures or class dismissals can cause substantial harm, such as negatively affecting education, social and emotional development, and physical and mental health of children and young persons (*2*,*3*). Hence, rigorous evaluation of public health effects of school-based measures are needed to guide disease control and prevention policies. We analyzed epidemiologic and school-reported data to determine the effects of school-based measures on COVID-19 transmission in Hong Kong during 2022.

## The Study

**Figure 1:**
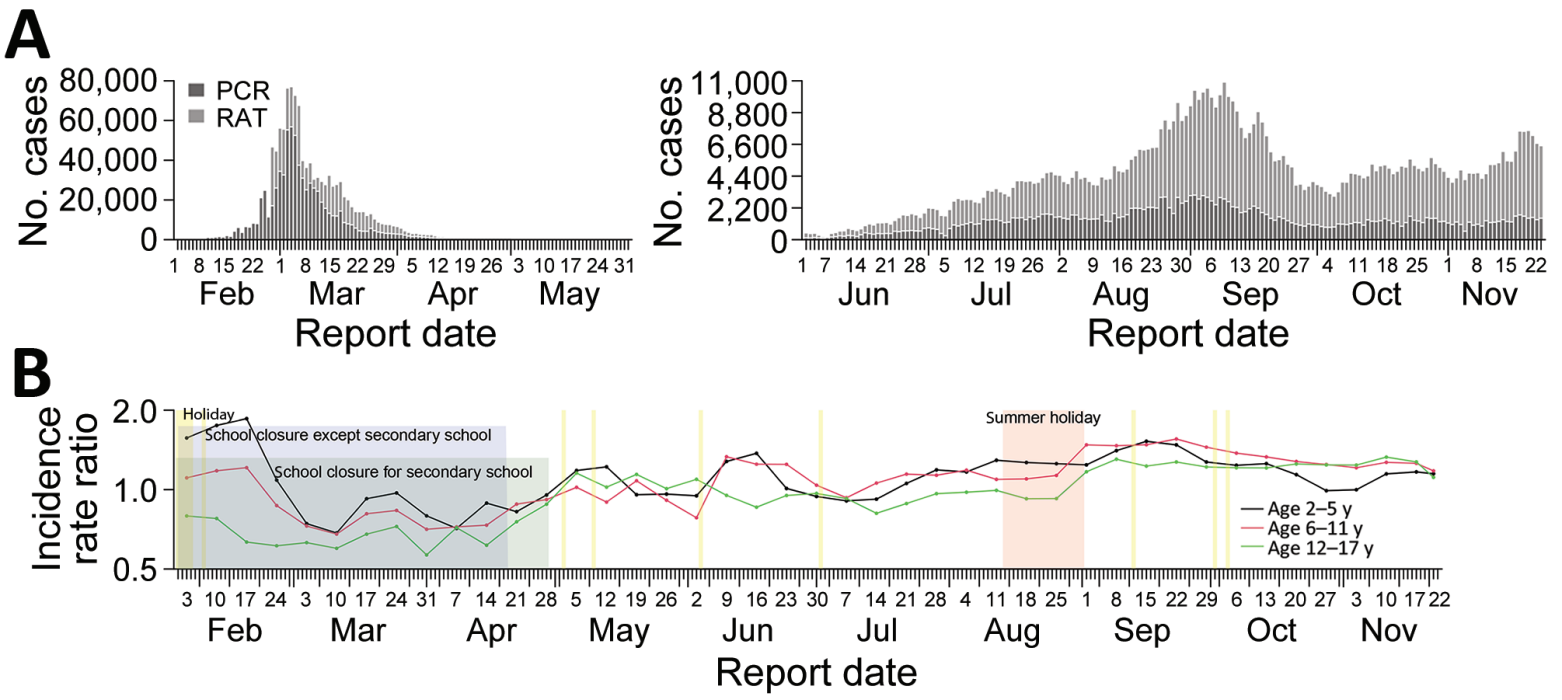
Epidemiology of 5th and 6th COVID-19 outbreak waves, Hong Kong, 2022, evaluated in study of effects of school-based preventive measures on COVID-19 incidence. A) Epidemic curves of 5th (February–April 2022, left) and 6th (beginning in June 2022, right) COVID-19 waves according to reporting date and test type. B) Incidence rate ratios of school-age children in kindergarten (age 2–5 y), primary schools (age 6–11 y), and secondary schools (age 12–17 y) in the 5th and 6th COVID-19 waves. Referent was adults (age >18 y). Yellow shading indicates a school holiday. RAT, rapid antigen test.

The study was approved by the Institutional Review Board of the University of Hong Kong. We analyzed COVID-19 data reported to the Hong Kong Centre for Health Protection that included PCR-confirmed cases during January 1–November 22, 2022, and RAT-confirmed cases during February 26–November 22, 2022. Confirmative PCR was administered for RAT-confirmed cases reported during June 7–August 28, 2022. We found that age-specific incidence rate ratios for infections in children compared with adults (>18 years of age) in the 6th wave were slightly higher than in the 5th wave (Figure 1).

We divided the study period into 3 segments: school closure, summer holiday, and normal school days (days other than closures and holidays). We stratified cases into 4 age groups: 2–5 years (preschool/kindergarten students), 6–11 years (primary school students), 12–17 years (secondary school students), and >18 years (adults). We used a Poisson generalized additive regression model, adjusting for time trend of COVID-19 cases and including the age groups and study periods (Appendix), to determine the effects of school closure and summer holiday on COVID-19 transmission in school-age children.

During normal school days (Table 2), the COVID-19 incidence rate for kindergarten students was 24% (95% CI 22%–25%), for primary school students was 34% (95% CI 32%–35%), and for secondary school students was 19% (95% CI 18%–20%) higher than for adults, suggesting that school-age children had a higher infection risk than adults during normal school days. During the 5th-wave school closure, the incidence rate for kindergarten students was 31% (95% CI 29%–32%), for primary students was 42% (95% CI 41%–43%), and for secondary students was 46% (95% CI 46%–47%) lower than for adults. During the summer holiday when most schools were closed during the 6th wave, the COVID-19 incidence rate for kindergarten students was 12% (95% CI 9%–15%), for primary students was 28% (95% CI 26%–30%), and for secondary students was 32% (95% CI 30%–34%) lower than for adults. Assuming that school-based interventions had no effect on adults, effectiveness of school closure on reducing COVID-19 transmission was 31%–46% during the 5th wave and 12%–32% during the 6th wave.

**Table 2:** Incidence rate and incidence rate ratio estimates according to the Poisson generalized additive regression model in study of effects of school-based preventive measures on COVID-19 incidence, Hong Kong, 2022*

Period and age group	Incidence rate†	IRR (95% CI)
Normal school days		
Age, y		
>18	169	Referent
2–5	204	1.24 (1.22–1.25)
6–11	220	1.34 (1.32–1.35)
12–17	196	1.19 (1.18–1.2)
School closure		
Age, y		
>18	727	Referent
2–5	622	0.69 (0.68–0.71)
6–11	560	0.58 (0.57–0.59)
12–17	461	0.54 (0.53–0.54)
Summer holiday		
Age, y		
>18	292	Referent
2–5	370	0.88 (0.85–0.91)
6–11	324	0.72 (0.7–0.74)
12–17	275	0.68 (0.66–0.7)

*Adjusted for the time trend of COVID-19 cases. IRR, incidence rate ratio. †Per 1,000 person-years.

**Figure 2:**
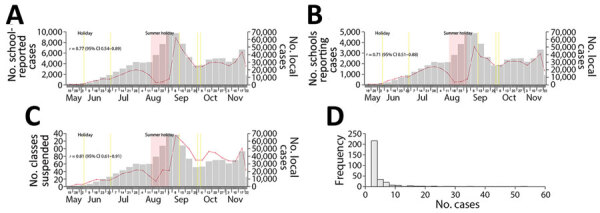
School-reported data during the 6th COVID-19 wave, Hong Kong, 2022, evaluated in study of effects of school-based preventive measures on COVID-19 incidence. A) Weekly numbers of school-reported cases during the 6th wave, beginning in June 2022. B) Weekly number of schools reporting >1 case during the 6th wave. C) Weekly number of class suspensions (classes with >2 COVID-19 cases) during the 6th wave. Yellow shading indicates school holiday; red shading indicates summer holiday. Pearson correlation coefficient *r* and 95% CI were calculated for data reported weekly. D) Distribution of 299 suspected school clusters of COVID-19 by size of cluster (no. cases). A school cluster was defined as a school that reported>2 cases within 7 days. Scales for the y-axes in panels A–C differ substantially to underscore patterns but do not permit direct comparisons.

We collected school-related data from daily press conferences and press releases, including numbers of school-reported cases (students and staff), class suspensions, and schools reporting >1 case during periods of in-person learning (Appendix). Excluding summer holidays, weekly case numbers in the community were highly correlated with weekly numbers of school-reported student and staff cases (Pearson correlation coefficient *r* = 0.77, 95% CI 0.54–0.89), schools reporting >1 case (*r* = 0.75; 95% CI 0.51–0.88), and >2 cases of class suspension (*r* = 0.81, 95% CI 0.61–0.91) (Figure 2). Among 299 suspected school clusters, defined as schools that reported>2 COVID-19 cases within 7 days, a total of 66 (22%) had >5 cases and 22 (7%) had >10 cases. The largest suspected cluster recorded 53 cases in a secondary school that had ≈750 students and ≈75 staff. The second-largest suspected cluster had 35 cases in an international school that had ≈960 primary and secondary students.

## Conclusions

We found that school-age children had a higher SARS-CoV-2 infection risk than adults in Hong Kong, consistent with another study suggesting that children were more susceptible to Omicron variants compared with adults (*4*). School closure and summer holiday effectively reduced incidence rates in school-age children during the 5th and 6th COVID-19 waves, aligning with modeling and simulation studies demonstrating the effectiveness of school closure in reducing COVID-19 transmission (*5*–*7*). We noted that the reduction in incidence rates for school-age children during school closure in the 5th wave was greater than that during the summer holiday in the 6th wave. Potential explanations for those results are that schools might not have been completely closed during summer holiday, possibly increasing the number of contacts between children; that Omicron BA.4/BA.5 variants were more prevalent during the 6th wave (Appendix Figure 1); or that higher ascertainment rates existed among students who had RAT used to detect COVID-19.

The strong positive correlation between school-reported data and community case numbers after school reopening indicated school reopening did not cause abnormal increases in community COVID-19 incidence. The largest suspected school cluster had 53 COVID-19 cases, comparable to other superspreading events, such as the 67-case cluster caused by Omicron BA.1 and 167-case cluster caused by Omicron BA.2 in January 2022 (*8*). Those results suggest that school reopening did not pose additional superspreading risks in school settings. 

The first limitation of our study is that some school-reported COVID-19 infections could have originated elsewhere in the community, such as at home, instead of in schools. Although students and staff were required to conduct daily rapid tests and report positive results to schools and the government, underreporting cannot be ruled out. Second, we extracted school outbreak data from press conferences; thus, some details could have been missed. Third, we cannot exclude the possibility that some schools did not fully adhere to guidelines, particularly regarding class size and lesson duration; however, we lacked school-level data to account for that possibility. Fourth, our analysis did not account for changes in dominant virus strains (Omicron BA.2 in the 5th wave, Omicron BA.5 in the 6th wave). Finally, we considered school-based measures as a collective package and were unable to determine individual effects of specific measures on COVID-19 transmission.

In summary, we evaluated school closure and school reopening accompanied by multilayer school-based preventive measures for COVID-19 in Hong Kong, which was informative as a guide for implementing and relaxing those measures. Our results might not be directly generalizable for other respiratory pathogens because of differences in transmission and intervention effectiveness. However, our results are consistent with modeling studies suggesting that safe school reopening is possible when appropriate alternative school-based preventive measures are used (*9*–*12*). If resurgence in case numbers or emergence of variants with higher transmissibility in children occurs, school closure remains an option to reduce transmission among children.

Appendix Additional information for effects of school-based preventive measures on COVID-19 incidence, Hong Kong, 2022.
