# Supplementary material for: Effects of School-Based Preventive Measures on COVID-19 Incidence, Hong Kong, 2022
Source: Emerg Infect Dis. 2023 Sep;29(9):1850–4. doi: 10.3201/eid2909.221897 (PMC10461670; doi:10.3201/eid2909.221897)
Supplement: Appendix — Additional information for effects of school-based preventive measures on COVID-19 incidence, Hong Kong, 2022. [file 22-1897-Techapp-s1.pdf]

Article DOI: https://doi.org/10.3201/eid2909.221897
EID cannot ensure accessibility for supplementary materials supplied by authors. Readers who have difficulty accessing supplementary content should contact the authors for assistance.

Effects of School-Based Preventive Measures on COVID-19 Incidence, Hong Kong, 2022

Appendix

Additional Methods

Data Extraction

School-reported data were extracted from daily press conferences during May 16–September 23, 2022. After September 23, 2022, daily press conference were canceled, and we extracted the relevant information from daily press releases. Age-specific case data were obtained from the Hong Kong Centre of Health Protection; data were available until November 22, 2022. Numbers of cases (without age information) were obtained from daily press releases until November 22, 2022. We obtained population incidence denominators for age groups from the Census and Statistics department of the Hong Kong Special Administrative Region government.

Poisson Generalized Additive Regression Model

To determine the effects of school closure on COVID-19 incidence rates in school-age children, we fitted a Poisson generalized additive regression model adjusting for time trend of cases. Denoting case numbers at week t for age group i (1 = adults $\geq$18 years of age, 2 = kindergarten students 2–5 years of age, 3 = primary students 6–11 years of age, 4 = secondary students 12–17 years of age) as X_{it}, population size of the age group i as P_{it}, and time period as T_i (1 = baseline period, 2 = school closure, 3 = summer holiday), we have

$$\log(X_{it}) = \log(P_{it}) + \beta_{11} \times I(i = 2) + \beta_{12} \times I(i = 3) + \beta_{14} \times I(i = 4) + \beta_{21} \times I(i = 2 \& t \in T_2) + \beta_{22} \times I(i = 3 \& t \in T_2) + \beta_{23} \times I(i = 4 \& t \in T_2) + \beta_{31} \times I(i = 2 \& t \in T_3) + \beta_{32} \times I(i = 3 \& t \in T_3) + \beta_{33} \times I(i = 4 \& t \in T_3).$$

The incidence rate ratio for kindergarten students is indicated by exponential of β_{11}, $\exp(\beta_{11})$, primary students by $\exp(\beta_{12})$, and secondary students by $\exp(\beta_{13})$ compared with adults in the baseline period. Compared with adults in school closure, the incidence rate ratio for kindergarten students is indicated by $\exp(\beta_{21})$, primary students by $\exp(\beta_{22})$, and secondary students by $\exp(\beta_{23})$. Compared with adults in summery holiday, the incidence rate ratio for kindergarten students is indicated by $\exp(\beta_{31})$, primary students by $\exp(\beta_{32})$, and secondary students by $\exp(\beta_{33})$.

Appendix Table 1. School-based control measures since 2022

Action date	Action	Announcement	Sources
2022 Jan 14	Primary schools, kindergartens, and kindergarten/child care centers should suspend face-to-face classes and on-campus activities by this Friday until their Lunar New Year holidays end	2022 Jan 11	https://www.news.gov.hk/eng/2022/01/20220111/20220111_104753_827.html?type=category&name=covid19&tl=t
2022 Jan 24	All secondary schools should suspend face-to-face classes and all on-campus activities on or before January 24 until their Chinese New Year holidays.	2022 Jan 20	https://www.news.gov.hk/eng/2022/01/20220120/20220120_122040_196.html?type = category&name = covid19&tl = t
2022 Feb 8	Schools across the city will continue to suspend face-to-face classes until February 21, 2022	2022 Jan 27	https://www.news.gov.hk/eng/2022/01/20220127/20220127_184401_063.html?type = category&name = covid19&tl = t
2022 Feb 14	All kindergartens, primary schools, secondary schools and tutorial schools will continue the suspension of face-to-face classes and on-campus activities until March 6.	2022 Feb 14	https://www.news.gov.hk/eng/2022/02/20220214/20220214_174151_936.html?type = category&name = covid19&tl = t
2022 Mar 1	Summer holiday start from March or April	2022 Feb 22	https://www.news.gov.hk/chi/2022/02/20220222/20220222_193732_757.html?type = category&name = covid19&tl = t
2022 Apr 19	Chief Executive Carrie Lam today announced that primary schools, international schools, and kindergartens may resume face-to-face classes as early as April 19 after the Easter holidays. Secondary schools may restart in-person classes after the examinations of the 2022 Hong Kong Diploma of Secondary Education Examination core subjects are completed.	2022 Mar 21	https://www.news.gov.hk/eng/2022/03/20220321/20220321_124717_054.html?type=category&name=covid19&tl=t
2022 Apr 19	The Government today announced that schools will resume face-to-face classes in phases after the Easter holidays. All school staff, teachers, and students are required to conduct COVID-19 rapid antigen tests (RATs) daily and only those who have obtained a negative result will be allowed to enter the schools.	2022 Apr 11	https://www.news.gov.hk/eng/2022/04/20220411/20220411_120620_439.html?type=category&name=covid19&tl=t
2022 May 19	The Education Bureau announced today the updated arrangements concerning antiepidemic measures in schools; it will relax conditions for students to participate in maskwearing activities on a half-day basis. Such activities include speech days, parents' days, open days, and campus visits. However, students must still follow antiepidemic rules while attending the events.	2022 May 19	https://www.news.gov.hk/eng/2022/05/20220519/20220519_174157_317.html?type=category&name=covid19&tl=t
2022 Jun 17	All teachers, school staff, and students have to complete an RAT every school day and obtain a negative test result before returning to school for work or lessons.	2022 Jun 17	https://www.news.gov.hk/eng/2022/06/20220617/20220617_150957_812.html?type = category&name = covid19&tl = t

Appendix Table 2. Vaccination requirements for entering schools during 2022*

Date	Focus	Age, y	School type	Action	Remarks	Sources
2022 Feb 24	Staff	$\geq$18	Kindergartens, primary and secondary schools, tutorial schools	From February 24, apart from those who are exempted, all teaching and nonteaching staff, people providing on-campus services and school visitors will be required to present vaccination records of at least the first COVID-19 vaccine dose before their entry into school premises. The above measures will still be enforced during suspension of face-to-face classes. For those exempted personnel, such as those who are unfit for vaccination due to health reasons, they are still required to conduct COVID-19 tests once every three days.	Announced on 2022 Jan 20. The arrangement does not apply to students.	https://www.info.gov.hk/gia/general/202202/14/P2022021400639.htm; https://www.news.gov.hk/eng/2022/01/20220120/20220120_194630_073.html
2022 Feb 24	Staff, students	$\geq$12	NA	From February 24, persons $\geq$12 years of age with $\geq$1 COVID-19 vaccination dose will be permitted to enter specified premises.	Announced on 2022 Jan 21.	https://www.news.gov.hk/eng/2022/02/20220221/20220221_153338_846.html

Date	Focus	Age, y	School type	Action	Remarks	Sources
2022 Apr 21	Staff	≥18	Kindergartens, primary and secondary schools, tutorial schools	Teachers and school staff directly employed by schools who have received only one dose should receive the second dose before April 21.	Announced on 2022 Jan 20. The arrangement does not apply to students.	https://www.news.gov.hk/eng/2022/01/20220120/20220120_194630_073.html
2022 Apr 30	Students	12–17	NA	During the second stage from April 30 to June 29, if persons 12–17 years of age have received their first dose of COVID-19 vaccine for <6 months, their vaccine pass remains valid. However, if they have received their first dose ≥6 months ago, they will need to receive a second dose to continue to use the vaccine pass.	Announced on 2022 Mar 4.	https://www.news.gov.hk/eng/2022/03/20220304/20220304_210043_224.html
2022 Jun 30	Students	12–17	NA	Starting from the third stage from June 30, if persons 12–17 years of age have received their second dose of COVID-19 vaccine <9 months ago, they can still use the Vaccine Pass. If they have received their second dose for >9 months ago, they are required to receive a third dose to continue to use the vaccine pass.	Announced on 2022 Mar 4.	https://www.news.gov.hk/eng/2022/03/20220304/20220304_210043_224.html
2022 Aug 5	NA	NA	NA	All teachers and school staff directly employed by the schools to meet the requirements of the Vaccine Pass, and ≥90% of the total number of students eligible for vaccination to receive 2 doses of vaccine >14 d apart.		https://www.news.gov.hk/eng/2022/08/20220805/20220805_164616_433.html
2022 Aug 5	Students	NA	NA	Secondary or primary school students who have received two jab doses >14 d apart may attend nonacademic extracurricular activities at school after lessons or during the other half-day of a school day.		https://www.news.gov.hk/eng/2022/08/20220805/20220805_164616_433.html
2022 Sep 30	Students	5–11	NA	Starting from September 30, children 5–11 years of age are required to have received at least the first dose within 3 months to comply with the vaccination requirements under the Vaccine Pass for entering specified premises (Note). If they received their first dose 3 months ago or earlier, they are required to receive the second dose to continue to be eligible to use the Vaccine Pass.	Announced on 2022 Sep 8	https://www.info.gov.hk/gia/general/202209/08/P2022090800481.htm?fontSize = 1
2022 Sep 30	NA	≥12	NA	Starting from September 30, the grace period for persons ≥12 years of age to receive the third dose after the second dose will be shortened from the original 6 months to 5 months. For those who had received the second dose within 5 months, their Vaccine Pass is still valid, but they must receive the third dose within 5 months after receiving the second dose (instead of the original 6 months) to continue using the Vaccine Pass afterwards.	Announced on 2022 Sep 8	https://www.info.gov.hk/gia/general/202209/08/P2022090800481.htm?fontSize = 1
2022 Nov 30	Students	5–11	NA	Starting from November 30, the vaccination requirement for all children 5–11 years of age under the Vaccine Pass will have to receive the second dose.	Announced on 2022 Sep 8	https://www.info.gov.hk/gia/general/202209/08/P2022090800481.htm?fontSize = 1
2022 Nov 30	NA	≥12	NA	Starting from November 30, persons ≥12 years of age are required to have received the third dose to meet the vaccination requirements under the Vaccine Pass.	Announced on 2022 Sep 8	https://www.info.gov.hk/gia/general/202209/08/P2022090800481.htm?fontSize = 1

*NA, not applicable.

Appendix Table 3. Summary of rapid tests available in Hong Kong and their sensitivity and specificity provided by manufacturer*

Brand name	Manufacturer	Web sites	Sensitivity, %	Specificity, %
AESKU.RAPID SARS-CoV-2	AESKU.Diagnostics	https://eshop.medifasthk.com/en/AESKU-COVID-19-Rapid-Antigen-Test-2-Tests	100	98.00
AESKU.RAPID SARS-CoV-2	AESKU.Diagnostics	https://www.aesku.com/index.php/diagnostics/aesku-rapid-sars-cov-2-antigen-tests	100	98
AESKU.RAPID SARS-CoV-2	AESKU.Diagnostics	https://www.aesku.com/index.php/diagnostics/aesku-rapid-sars-cov-2-antigen-tests	100	98
Aichek, COVID-19 Antigen Home Test	Hangzhou Aichek Medical Technology	https://www.consumer.org.hk/tc/rapid_antigen_test_search	NA	NA
FIATest, SARS-CoV-2 Antigen Rapid Test Cassette(Nasopharyngeal Swab)	Hangzhou All Test Biotech	https://www.custom-monoclonalantibody.com/sale-2991704-high-quality-microalbumin-test-use-by-fiatest-fluorescence-immunoassay-analyzer-in-human-whole-blood.html	NA	NA
ALLTest, SARS-CoV-2 Antigen Rapid Test (Nasal Swab) (INCP-502-N)	Hangzhou AllTest Biotech	https://www.maskwholesale.eu/rapid-tests/alltest-corona-antigen-nasal-swab-rapid-test_292_1314/	95.40	99.40
Biosynex Autotest Antigenique COVID-19 Ag	Biosynex	https://www.biosynex.com/products/pharmacy/diagnosis/self-tests/biosynex-covid-19-ag-bss-self-test/?lang=en	97.20	100
BioTeke SARS-CoV-2 Antigen Test Kit	BioTeke Corporation (Wuxi)	https://www.hktvmall.com/hktv/en/main/Milan-Store/s/H0257001/Personal-Care-%26-Health/Personal-Care-%26-Health/Health/Self-Test-Kits/BIOTEKE-Covid19-Test-Kit-SARSCoV2-Antigen-Rapid-Test-Kit-Omicron-Delta-3pc-Random-Box/p/H0257001_S_BIOTEKE-3?scrollTo=descriptionsTab	95	99.28
BIOUHAN, SARS-CoV-2 Antigen Rapid Test (Colloidal Gold Method)	Biohit Healthcare (Hefei)	https://www.biouhan.com/AgCC/index.aspx	96.12	99.49
Clungene, COVID-19 Antigen Rapid Test	Hangzhou Clongene Biotech	https://www.hansagt24.com/en/covid-19-antigen-rapid-test-for-professional-use/80-clungene-covid-19-antigen-rapid-test-cassette.html	98	99.70
CO-Check SARS-CoV-2 Antigen LFIA Test	Sanwa BioTech (三和生物科技)	https://www.tga.gov.au/resources/covid-19-test-kits/co-check-sars-cov-2-antigen-lfia-test-poct	NA	NA
Coretests, COVID-19 Ag Test	Core Technology	https://patika1.hu/CoreTest-COVID-19-Antigen-teszt-orr-1x	95.11	100
Coronavirus (2019-nCoV)-Antigentest-	Beijing Hotgen Biotech	http://www.hotgen.com.cn/zl1.html	90.70	100
Hotgen Novel Coronavirus 2019 n-CoV Antigen Test (for self-testing use)	Beijing Hotgen Biotech	https://www.drb-operations.de/en/hotgen-coronavirus-rapid-test/	95.37	99.13
COVID-19 / influenza A virus / influenza B virus Antigen test kit (colloidal gold)	Xiamen Hopegen Medical Technology	https://www.shoptest.com/covid19-test-hopegen321.html	96	99
Covid-19 Antigen CARD Kit	GeFosun Diagnostics (Shanghai)	https://eshop.pasioncare.com/en/products/fosun-diagnostics-covid-19-antigen-card-kit-2	96.75	98.26
COVID-19 Antigen Nasal Test Kit	Assure Tech. (Hangzhou)	https://www.assuretech-product.com/search/COVID.html	96.80	99.80
COVID-19 Antigen Rapid Detection Kit (Colloidal Gold)	Pro-med (Beijing) Technology	https://www.consumer.org.hk/tc/rapid_antigen_test_search	NA	NA
COVID-19 Antigen Rapid Test	Beijing North Institute of Biotechnology	https://www.consumer.org.hk/tc/rapid_antigen_test_search	94.70	99
COVID-19 Antigen Rapid Test	Shenzhen Everbest Machinery Industry	https://cem-instruments.de/products/covid-19-antigen-schnelltest/	92.73	100
COVID-19 Antigen Rapid Test Kit	Beijing Kewei Clinical Diagnostic Reagent	http://en.keweidiagnostic.com/index.php?m = content&c = index&a = show&catid = 200&id = 184	96.18	100
COVID-19 Antigen Rapid Test Kit (Colloidal Gold)	AmonMed	http://en.amonmed.com/	95.05	99.55

Brand name	Manufacturer	Web sites	Sensitivity, %	Specificity, %
COVID-19 Antigen Rapid Test Kit (Colloidal gold)	Xiamen AmonMed Biotechnology	https://www.consumer.org.hk/tc/rapid_antigen_test_search	100	99.55
FIATest, COVID-19 Antigen Test Cassette	Hangzhou AllTest Biotech	https://www.alltests.com.cn/Home/ProductInfo/385	NA	NA
Flowflex SARS-CoV-2 Antigen Rapid Test	Acon Biotech (Hangzhou)	https://www.aconbio.com/en/ACON/Rapidtest/	97.22	99.71
fluorecare, SARS-CoV-2 Antigen Test Kit (Colloidal Gold Chromatographic Immunoassay)	Shenzhen Microprofit Biotech	https://www.sasa.com.hk/SalePage/Index/268951	92.93	100
Fosun Covid-19 Ag CARD	Fosun Diagnostics (Shanghai)	https://eshop.pasioncare.com/en/products/fosun-diagnostics-covid-19-antigen-card-kit-2	96.75	98.26
Hecin, 2019-nCoV Antigen Test Kit (colloidal gold method)	Guangdong Hecin	https://hplushk.com/products/hecin	97.09	99.78
iClean, COVID-19 Ag Rapid Test Kit (Colloidal Gold)	Huachenyang (Shenzhen) Technology	https://www.linkedin.com/pulse/iclean-covid-19-antigen-rapid-test-listed-eu-common-list-ivy-peng/	91	100
INDICAID COVID-19 Rapid Antigen Test	Phase Scientific	https://www.intecasi.com/rapid-sars-cov-2-antigen-test-nasal-swab-for-self-testing-use_p55.html	93.64	100
Medomics, SARS-CoV-2 Antigen Rapid Test (LFIA)	Jiangsu Medomics Medical Technology	https://www.medomics-dx.net/product/SARS-CoV-2-Antigen-Test-Kit-%28LFIA%29-Home-Use-Single-Pack-492.html	97.73	99.51
Novel Coronavirus (SARS-Cov-2) Antigen Rapid Test Device (nasal swab)	Hangzhou Realy Tech	https://www.takemycovidtest.com.au/products/p/covid-19-rapid-home-test-kit-1-pack-sxr9t	95.38	99.99
One Step Test for SARS-CoV-2 Antigen (Colloidal Gold)	GETEIN Biotech	https://leo-shop.de/produkt/getein-schnelltest/	97.06	98.71
GP One Step Test for SARS-CoV-2 Antigen	GETEIN Biotech	http://www.medical.iap.com.hk/index.php?route=product/product&product_id=196	97	98.7
Panbio COVID-19 Antigen Self-Test	Abbott Rapid Diagnostics	https://www.globalpointofcare.abbott/ww/en/products-solutions.html	98.10	100
Rapid COVID-19 Antigen Self-Test	Healgen Scientific	https://www.quadratech.co.uk/product/healgen-rapid-covid-19-antigen-self-test-single-cassette-ce-marked-15min-nasal-swab/	97.25	98.73
Rapid SARS-CoV-2 Antigen Test	InTec PRODUCTS	https://www.intecasi.com/rapid-sars-cov-2-antigen-test-nasal-swab-for-self-testing-use_p55.html	93.64	100
RightSign, COVID-19 Antigen Rapid Test Cassette (Nasal Swab)	Hangzhou Biotest Biotech	https://hygiene100.de/produkt/rightsign-hangzhou-biotest-biotech-covid-19-antigen-rapid-test-cassette-nasal-swab/	93.75	99.12
Roche, SARS-CoV-2 Antigen Self Test Nasal	SD. Biosensor	https://diagnostics.roche.com/gb/en/products/params/sars-cov-2-antigen-self-test-nasal.html#productSpecs	91.10	99.60
Roche, SARS-CoV-2 Rapid Antigen Test Nasal	SD. Biosensor	https://diagnostics.roche.com/global/en/products/params/sars-cov-2-rapid-antigen-test.html	95.50	99.20
YHLO GLINE-2019-nCoV Ag	Shenzhen Watmind Medical	https://www.mblbio.com/e/products/ivd/list/YH-G86247E.html	96.93	99.25
SARS-CoV-2 Ag Self-Test Kit (Nasal Swab)	Shenzhen Watmind Medical	https://www.hktvmall.com/hktv/en/main/SUN-TONE-RETAIL-SOLUTIONS-LIMITED/s/H8612001/Personal-Care-%26-Health/Personal-Care-%26-Health/Health/Self-Test-Kits/COVID-SelfTest-Kit-Nasal-Swab-1-kit-15minTEST-RATtest-Omicron-Delta-CE-Ctsensitivity/p/H8612001_S_AQ_Adult_1kit	95.70	100
SARS-CoV-2 and Influenza A+B Antigen Combo Rapid Test (Nasal Swab)	Hangzhou AllTest Biotech	https://www.alltests.com.cn/Home/product	97.60	99.70
SARS-CoV-2 Antigen Assay Kit (Collodial Gold Method)	Zybio	https://mybio.ie/products/zybio-sars-cov-2-antigen-assay-kit-home-self-test-188-unit-case-ce-ivd-certified	88.79	99.04
SARS-CoV-2 Antigen Rapid Test Cassette	Hangzhou Sejoy	https://rapidtest-lab.com/en/termek/sejoy-sars-cov-2-antigen-nasal-rapid-test/	97.9	99.99

Brand name	Manufacturer	Web sites	Sensitivity, %	Specificity, %
SARS-CoV-2 Antigen Rapid Test Device	Zhuhai Encode Medical Engineering	https://www.consumer.org.hk/tc/rapid_antigen_test_search	95	100
SARS-CoV-2 Antigen Rapid Test Kit	Labnovation	https://www.consumer.org.hk/tc/rapid_antigen_test_search	94	100
SARS-CoV-2 Antigen Rapid Test Kits for Self-testing (Colloidal Gold Immunochromatography)	Beijing Lepu Medical Technology	https://en.lepumedical.com/lepu-medicals-sars-cov-2-antigen-rapid-test-acquir.html	95.90	100
SARS-CoV-2 Virus Antigen Detection Kit (colloidal gold method)	BGI	NA	NA	NA
Savewo, COVID-19(SARS-CoV-2) Antigen Test Kit (Colloidal Gold)	Anhui Deepblue Medical Technology	https://www.mdd.gov.hk/tc/whats-new/rapid-antigen-tests-covid-19/index.html#gallery_tb1_3-2	96.40	99.80
Skypro, Rapid COVID-19 Antigen Test (Colloidal Gold)/ Nasal Swab	Anbio (Xiamen) Biotechnology	https://skypro.com.hk/?p = 5962&lang = en	98	100
Verino Pro SARS-CoV-2 Ag Rapid Test	VivaChek Biotech (Hangzhou)	https://www.anjoycycle.com/en/products/verino-pro-rapid-antigenic-test-sars-cov-2-ag	97.42	99.99
Wondfo, Wondfo 2019-nCoV Antigen Test (Lateral Flow Method)	Guangzhou Wondfo	https://www.abingdonhealth.com/products/covid-19-antigen-self-test/	91.63	99.84
YHLO, GLINE-2019-nCoV Ag	Shenzhen YHLO Biotech	https://www.lyreco.com/webshop/ENHK/yhlo-gline-2019-ncov-ag-rapid-antigen-test-single-test-product-000000000016214462.html	96.38	99.56
Dynamiker SARS-CoV-2 Ag Rapid Test	Dynamiker Biotechnology (Tianjin) Co.,Ltd	https://covid-19-diagnostics.jrc.ec.europa.eu/devices/detail/2533	95.7	99.1
Jinwofu Novel Coronavirus (SARS-CoV-2) Antigen Rapid Test Kit	Jinwofu Bioengineering Technology Co., Ltd	https://bjjwf.cn/en/h-pd-36.html	96.71	99.73
Roche SARS-CoV-2 Rapid Antigen Test Nasal	SD Biosensor	https://diagnostics.roche.com/global/en/products/params/sars-cov-2-rapid-antigen-nasal-test.html#productSpecs	89.6	99.1
Roche SARS-CoV-2 Antigen Self Test Nasal	SD Biosensor	https://diagnostics.roche.com/global/en/products/params/sars-cov-2-antigen-self-test-nasal.html	95.8	100

*NA, not available.

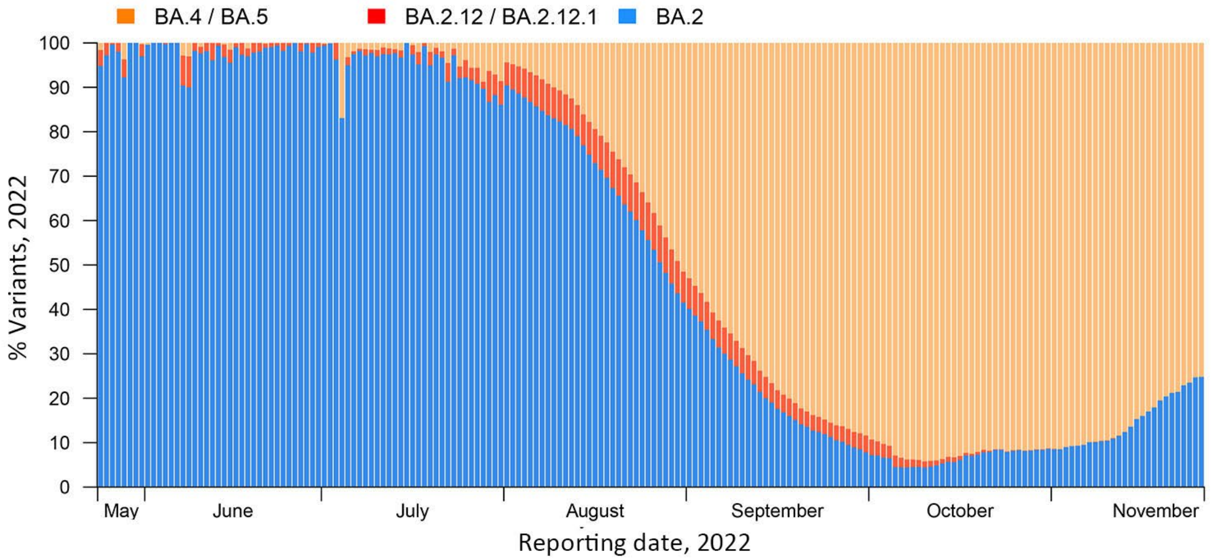

Appendix Figure 1. Percentages of SARS-CoV-2 Omicron BA.2 and BA.4/BA.5 variants during the 6th wave of the COVID-19 outbreak in Hong Kong in 2022.
